# Supplementary material for: Is domestic agricultural production sufficient to meet national food nutrient needs in Brazil?
Source: PLoS One. 2021 May 20;16(5):e0251778. doi: 10.1371/journal.pone.0251778 (PMC8136643; doi:10.1371/journal.pone.0251778)
Supplement: S1 Table — The ratio of Oil and Ethanol production to “Processed” crop (e.g. soybeans or sugar cane) from FBS was used to convert raw crop calories and nutrients to Oil or Ethanol. In the case of soybeans, the range of fraction of crop production that is converted to Oil is between 0.17 and 0.20 in the series, while the fraction of sugar cane ranges from 0.10 to 0.13. Once the “Processed” portions were calculated, they were allocated to “Export”, “Feed” and “Food” according to the Oil and Sugar sheets. (PDF) [file pone.0251778.s001.pdf]

FBS	Product (PAM/PPM)	Allocated to:	Observation
Other Uses	Cottonseed	Fibres	
	Potatoes	Food	Staple food in Brazil. Other uses represent 2% of the production in the period
	Cassava	Food	Staple food in Brazil. Other uses represent 6% of the production in the period
	Milk	Food	Cheese, yoghurt, and other dairy products for human consumption
	Sugar Cane	Ethanol for biofuel	
Processed	Groundnut	Proportionally as food or feed, according to the domestic use.	
	Rice	Proportionally as food or feed, according to the domestic use.	
	Barley	Proportionally as food or feed, according to the domestic use.	
	Coconuts	Proportionally as food or feed, according to the domestic use.	
	Maize	Proportionally as food or feed, according to	

		the domestic use.	
	Grapes	Proportionally as food or feed, according to the domestic use.	
	Sugar Cane	Food	Matches national sugar production.
