# Supplementary material for: Is domestic agricultural production sufficient to meet national food nutrient needs in Brazil?
Source: PLoS One. 2021 May 20;16(5):e0251778. doi: 10.1371/journal.pone.0251778 (PMC8136643; doi:10.1371/journal.pone.0251778)
Supplement: S2 Table — Data is shown for each 10 years, including future projections (2020–2060), to enhance visualization. RDA and AI values were taken from the United States Institute of Medicine and population projections from IBGE [46–48]. (PDF) [file pone.0251778.s002.pdf]

	1990	2000	2010	2020	2030	2040	2050	2060
CHO (g/d)	129.1	129.3	129.4	129.5	129.5	129.6	129.6	129.6
Fiber (g/d)	27.9	28.3	28.4	28.3	28.1	27.9	27.7	27.6
Proteins (g/d)	44.3	46.1	48.1	49.2	49.8	50.5	50.9	51.2
Vit. A (µg RAE/d)	472.1	486.3	501.5	509.0	514.3	519.6	522.7	524.5
Vit. C (µg/d)	64.1	66.7	69.7	71.5	72.5	73.6	74.3	74.7
Thiamin (mg/d)	0.98	1.00	1.03	1.05	1.06	1.07	1.07	1.08
Riboflavin (mg/d)	1.01	1.04	1.07	1.09	1.10	1.11	1.11	1.12
Niacin (mg/d)	12.8	13.2	13.5	13.7	13.8	14.0	14.0	14.1
Vit. B6 (mg/d)	1.12	1.16	1.21	1.25	1.27	1.30	1.33	1.34
Folate (µg/d)	338.3	348.3	358.9	364.3	367.9	371.7	373.8	375.1
Vit. B12 (µg/d)	2.03	2.09	2.15	2.18	2.20	2.23	2.24	2.25
Ca (mg/d)	828.3	833.0	836.2	830.6	835.2	840.5	844.5	850.8
Cu (mg/d)	0.76	0.78	0.80	0.82	0.82	0.83	0.84	0.84
Fe (mg/d)	11.0	11.1	11.1	10.9	10.7	10.5	10.3	10.2
Mg (mg/g)	295.2	306.7	318.5	324.9	329.4	333.8	336.4	337.9
Mn (mg/d)	1.83	1.86	1.89	1.91	1.93	1.94	1.95	1.95
P (mg/d)	765.6	770.4	764.0	748.6	745.3	742.2	737.6	735.8
Zn (mg/d)	8.18	8.39	8.62	8.71	8.79	8.87	8.91	8.93
K (mg/d)	2681.8	2721.5	2772.7	2802.1	2820.9	2838.6	2850.1	2856.7
