# Supplementary material for: Is domestic agricultural production sufficient to meet national food nutrient needs in Brazil?
Source: PLoS One. 2021 May 20;16(5):e0251778. doi: 10.1371/journal.pone.0251778 (PMC8136643; doi:10.1371/journal.pone.0251778)
Supplement: S3 Table — (PDF) [file pone.0251778.s003.pdf]

| | | BAU | No Beef | Ovo-lacto | Vegan |
|---|---|---|---|---|---|
| Nutrients from | Crops | All food nutrients | All food nutrients | All food crops nutrients plus a fraction of feed that would not be used for pigmeat and poultry neither exported | All food crops nutrients plus the fraction of feed proportionally allocated as food |
| | Eggs | All food nutrients | All food nutrients | All food nutrients | None |
| | Milk | All food nutrients | All food nutrients | All food nutrients | None |
| | Beef | All food nutrients | None | None | None |
| | Poultry | All food nutrients | All food nutrients | None | None |
| | Pigmeat | All food nutrients | All food nutrients | None | None |
| Land use | Food crops | Current land use | Current land use | Current land use | Current land use |
| | Feed | Current land use | Current land use | Current land use | Current land use |
| | Pasture | Current land use | Pasture for dairy production calculated as three times the number of milked cows multiplied by the average number of | Pasture for dairy production, calculated as three times the number of milked cows multiplied by the average number of | None |

| | | | | | |
|---|---|---|---|---|---|
| | | | cattle per hectare | cattle per hectare | |
