# Supplementary material for: Is domestic agricultural production sufficient to meet national food nutrient needs in Brazil?
Source: PLoS One. 2021 May 20;16(5):e0251778. doi: 10.1371/journal.pone.0251778 (PMC8136643; doi:10.1371/journal.pone.0251778)
Supplement: S4 Table — Data is shown for each 10 years, including future projections (2020–2060), to enhance visualization. The production of 2017 is the baseline for future projections, considering population dynamics and any agricultural expansion. (PDF) [file pone.0251778.s004.pdf]

	Diet scenario	1990	2000	2010	2020	2030	2040	2050	2060
kcal	BAU	105.9	119.5	127.5	120.0	113.0	109.6	109.1	111.3
	No Beef	103.1	116.3	123.0	115.5	108.8	105.5	105.0	107.2
	Ovo-lacto	118.8	135.3	148.7	147.5	137.8	133.6	133.0	135.8
	Vegan	134.2	150.0	164.7	165.6	154.7	150.0	149.4	152.4
CHO	BAU	463.6	492.2	460.7	439.7	413.8	401.1	399.3	407.3
	No Beef	463.3	491.9	460.2	439.2	413.4	400.7	398.9	406.9
	Ovo-lacto	463.3	491.9	460.2	439.2	413.4	400.7	398.9	406.9
	Vegan	638.3	732.5	759.7	773.5	728.0	705.6	702.4	716.6
Fiber	BAU	140.8	158.9	145.6	147.3	139.5	136.2	136.6	139.9
	No Beef	140.7	158.8	145.5	147.2	134.4	136.1	136.5	139.8
	Ovo-lacto	140.7	158.8	145.5	147.3	139.4	136.1	136.5	139.8
	Vegan	215.7	251.4	282.7	316.8	300.0	292.9	293.7	300.8
Proteins	BAU	233.2	272.3	296.1	312.9	290.6	277.9	274.4	278.6
	No Beef	217.4	254.8	272.6	290.1	269.5	257.6	254.4	258.4
	Ovo-lacto	205.2	227.9	234.1	246.3	228.8	218.7	216.0	219.3
	Vegan	266.9	308.3	397.3	451.3	419.2	400.8	395.7	401.9
Vit A	BAU	98.0	109.7	128.4	128.4	119.7	114.8	113.7	115.6
	No Beef	97.8	109.4	128.0	128.0	119.3	114.5	113.3	115.2
	Ovo-lacto	96.2	105.4	122.4	121.5	113.3	108.7	107.6	109.4
	Vegan	45.0	45.7	40.1	47.0	43.8	42.0	41.5	42.8
Vit. C	BAU	592.4	756.1	305.7	221.9	206.0	196.8	194.2	197.1
	No Beef	592.4	756.1	305.4	221.9	206.0	196.8	194.2	197.1
	Ovo-lacto	592.0	755.5	304.6	221.0	205.1	196.0	193.3	196.3
	Vegan	671.5	835.6	396.5	300.6	279.0	266.6	263.0	267.0
Thiamin	BAU	>1000	>1000	>1000	>1000	>1000	>1000	>1000	>1000
	No Beef	>1000	>1000	>1000	>1000	>1000	>1000	>1000	>1000
	Ovo-lacto	>1000	>1000	>1000	>1000	>1000	>1000	>1000	>1000
	Vegan	>1000	>1000	>1000	>1000	>1000	>1000	>1000	>1000
Riboflavin	BAU	>1000	>1000	>1000	>1000	>1000	>1000	>1000	>1000
	No Beef	>1000	>1000	>1000	>1000	>1000	>1000	>1000	>1000
	Ovo-lacto	>1000	>1000	>1000	>1000	>1000	>1000	>1000	>1000
	Vegan	>1000	>1000	>1000	>1000	>1000	>1000	>1000	>1000
Niacin	BAU	>1000	>1000	>1000	>1000	>1000	>1000	>1000	>1000
	No Beef	>1000	>1000	>1000	>1000	>1000	>1000	>1000	>1000
	Ovo-lacto	>1000	>1000	>1000	>1000	>1000	>1000	>1000	>1000

	Vegan	>1000	>1000	>1000	>1000	>1000	>1000	>1000	>1000
Vit. B6	BAU	>1000	>1000	>1000	>1000	>1000	>1000	>1000	>1000
	No Beef	>1000	>1000	>1000	>1000	>1000	>1000	>1000	>1000
	Ovo-lacto	>1000	>1000	>1000	>1000	>1000	>1000	>1000	>1000
	Vegan	>1000	>1000	>1000	>1000	>1000	>1000	>1000	>1000
Folate	BAU	244.4	273.8	285.7	302.9	282.4	271.0	268.3	272.8
	No Beef	244.0	273.4	285.0	302.3	281.8	270.5	267.8	272.3
	Ovo-lacto	243.7	272.7	284.1	301.2	280.8	269.5	266.8	271.3
	Vegan	343.6	390.9	523.5	584.6	545.0	523.1	517.8	526.6
Vit. B12	BAU	193.6	249.6	364.9	393.0	366.5	351.7	348.2	354.1
	No Beef	153.1	204.3	303.3	333.0	310.5	298.0	295.0	300.0
	Ovo-lacto	65.0	72.7	95.8	99.1	92.3	88.6	87.7	89.2
	Vegan	4.2	4.0	7.0	10.3	9.6	9.2	9.1	9.2
Ca	BAU	125.9	147.6	153.5	159.6	149.5	144.0	142.7	144.6
	No Beef	125.3	147.0	152.6	158.7	148.6	143.2	141.9	143.7
	Ovo-lacto	124.8	145.9	151.0	153.9	146.9	141.6	140.3	142.1
	Vegan	108.0	128.6	156.5	171.9	161.0	155.1	153.7	155.7
Cu	BAU	203.3	252.6	239.5	215.0	200.5	192.4	190.5	193.8
	No Beef	201.2	250.2	236.2	211.8	197.5	189.6	187.7	190.9
	Ovo-lacto	180.9	200.0	188.6	158.1	147.5	141.5	140.1	142.5
	Vegan	219.2	263.7	233.7	209.2	195.0	187.2	185.3	188.5
Fe	BAU	353.4	393.6	418.4	451.7	434.0	430.4	436.9	450.4
	No Beef	348.4	388.0	410.4	443.8	426.4	422.8	429.2	442.5
	Ovo-lacto	347.2	384.9	405.9	438.5	421.3	417.8	424.0	437.2
	Vegan	441.2	521.8	717.0	802.6	771.1	764.7	776.1	800.2
Mg	BAU	307.8	330.2	339.7	343.5	319.1	305.3	301.7	306.4
	No Beef	305.7	327.9	336.6	340.5	316.3	302.6	299.0	303.7
	Ovo-lacto	298.9	316.2	319.0	320.6	297.8	284.9	281.6	286.0
	Vegan	411.3	452.8	558.3	593.0	550.8	527.0	520.7	528.9
Mn	BAU	403.4	440.0	379.9	376.7	352.4	339.5	336.8	342.8
	No Beef	403.3	439.9	379.8	376.6	352.3	339.4	336.6	342.7
	Ovo-lacto	403.2	439.5	379.2	376.0	351.7	338.8	336.1	342.1
	Vegan	459.8	542.6	504.1	512.2	479.0	461.5	457.8	466.0
P	BAU	255.7	297.3	330.4	357.9	338.5	329.6	330.2	337.7
	No Beef	248.5	289.0	318.8	346.1	327.4	318.8	319.5	326.6
	Ovo-lacto	243.7	276.2	299.9	323.6	306.1	298.0	298.5	305.4

	Vegan	308.3	363.5	490.2	571.7	540.7	526.5	527.4	539.5
Zn	BAU	188.2	211.5	230.3	238.9	233.0	214.3	212.4	216.1
	No Beef	172.9	194.4	206.9	216.1	201.7	193.9	192.1	195.5
	Ovo-lacto	169.7	185.9	195.0	202.2	188.3	181.5	179.8	183.0
	Vegan	208.8	238.6	310.5	342.7	319.9	307.5	304.7	310.0
K	BAU	205.7	241.4	232.0	236.3	221.1	213.0	211.2	215.0
	No Beef	202.0	237.3	226.3	230.8	215.8	208.0	206.2	209.9
	Ovo-lacto	200.2	232.5	2019.5	222.8	208.5	200.9	199.2	202.8
	Vegan	257.5	300.1	358.5	387.9	362.9	349.6	346.7	352.9
