# Supplementary figures and images for: Is domestic agricultural production sufficient to meet national food nutrient needs in Brazil?
Source: PLoS One. 2021 May 20;16(5):e0251778. doi: 10.1371/journal.pone.0251778 (PMC8136643; doi:10.1371/journal.pone.0251778)

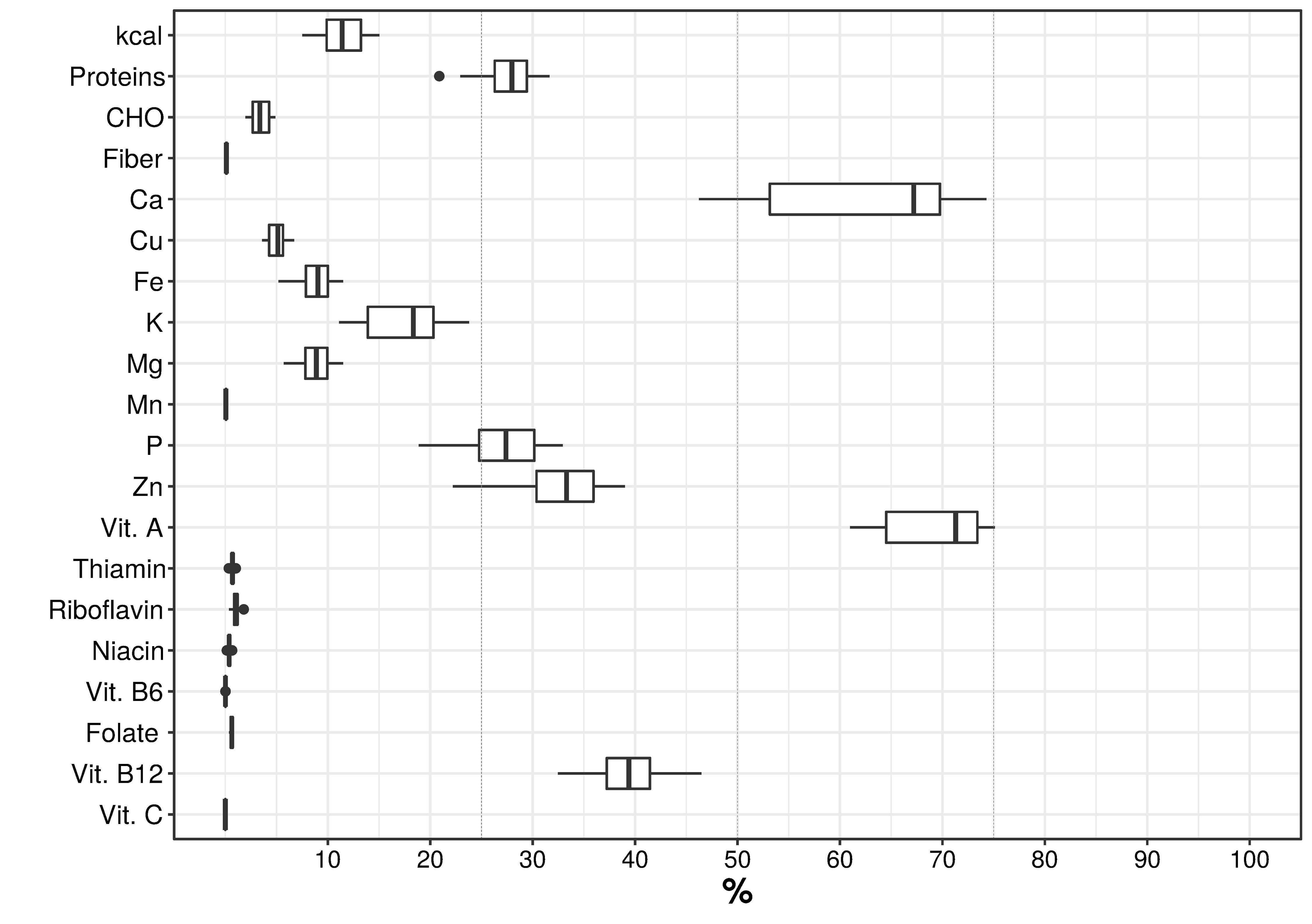

Supplement: S1 Fig — Boxplots indicate the contribution of pasturelands (cattle meat and milk) for food calories and nutrients, in relation to the total food production in 30 years. In most years of the analysis, only calcium and vitamin A were not mostly produced from crops. Except for these nutrients, along with zinc and vitamin B12 (~30% to ~45% produced from pastures), more than 70% of the nutrients and calories are produced from food crops. In spite of that, pastures occupy most farmlands in Brazil. (TIF) [file pone.0251778.s005.tif]
