# Supplementary material for: Is domestic agricultural production sufficient to meet national food nutrient needs in Brazil?
Source: PLoS One. 2021 May 20;16(5):e0251778. doi: 10.1371/journal.pone.0251778 (PMC8136643; doi:10.1371/journal.pone.0251778)
Supplement: Attachment — Submitted filename: Response to Reviewers.doc [file pone.0251778.s006.doc]

**RESPONSE TO REVIEWERS**

*Reviewer #1*

General comments

The paper rightly notes the important tradeoff between agricultural production and environmental impacts, and provides a detailed and spatially disaggregated analysis of domestic production and nutrient availability in Brazil. However, the paper misses important distinctions between nutrient needs, food security, and food demand, as well as key drivers of the latter (including income and preferences). If the authors’ intent is to focus on the potential to reduce agriculture’s environmental footprint in Brazil through alternative diets (as seems to be the case), this should be made more explicit in the title. If, however, the intent is to assess food security (as suggested in the current title), much more attention is needed on income, consumer preferences, trade, and other economic factors.

**Response**: Thanks for your careful evaluation of our work. We do agree with the reviewer that a complete assessment of food security requires addressing the broader aspects of food processing, retailing and, more importantly, access to healthy food. We thus changed the title and considered the suggestions made by the reviewer, as follows in the specific comments.


Specific comments

-Page 1, Title: food security has multiple dimensions (as the authors note on page 8), not all of which are examined in this paper, so a more accurate title would be “Is domestic agricultural production enough to meet national nutrient needs in Brazil?” (or “How much would alternative diets reduce agriculture’s environmental footprint in Brazil?”). At a minimum, the authors should note earlier in the paper that they focus on nutrient availability, and are using the term food security in that restricted sense.

**Response**: We changed the title to “Is domestic agricultural production sufficient to meet national food nutrient needs in Brazil?” and highlighted in the Introduction section the focus on food nutrients availability at these parts:

“This resulted in increasing deforestation trends since 2012, reaching in 2020 its highest peak since 2008 [27–29]. Political efforts to reduce environmental protection in Brazil were often based on the argument that more land is needed to produce food for the Brazilian population [30-32]. This argument was central to the debate that changed the environmental legislation in 2012 and is still argued in some proposals, both from the national congress and government, for promoting the agricultural expansion [30]. However, the proportion of land needed in the territory for meeting the nutritional food demand of the Brazilian population, currently around 210 million people, is unknown.”

“Shifting diets towards those which contain lower quantities of livestock products could reduce the amount of land needed to produce food.”

-Page 1, Abstract: the paper’s main question is posed in terms of environment (land) and food security, but needs better motivation for the authors’ focus on domestic production.

**Response**: We have rewritten the abstract

-Page 2, Line 13: trade is acknowledged as important for global food security, i.e. not only for Brazil but for countries that import from Brazil. But export earnings are not among the outcomes considered later in the paper alongside the environmental footprint of domestic production.

**Response**: Addressed in the discussion section.

“In this sense, [79] showed that agricultural expansion for export could improve the development indexes due to income and labour generation mainly in the Brazilian municipalities at the agricultural frontiers. Also, [80] claim that the expansion and improvement of this sector has generated substantial improvements in well-being throughout Brazil in several ways, such as governmental revenues for welfare programs and increasing private purchasing power. However, both works acknowledge that this is accompanied by income and land concentration, displacement of peasants and rural to urban migration with consequent losses of livelihoods when people move to marginal conditions. While the earnings from commodity exports improve local food security by increasing the economic affordability of food to more employed families, the benefits of global trade do not always necessarily overcome the social impacts. A socially-inclusive strategy and better multisectoral planning is needed for reducing exclusion in agricultural consolidated and under expansion, areas.”

-P2, L25: need to distinguish local/national environmental impacts (e.g. soil erosion, water quality) vs those with global implications (e.g. greenhouse gas emissions). The asserted link between land use change and negative impacts on agricultural yields also needs further explanation and support.

**Response**: Addressed in the text.

“Thus, additional large-scale land use changes are likely to cause negative environmental impacts with global implications for climate change via GHG emissions. Soil erosion, nutrient loss, water depletion and biodiversity loss [25, 74] are important regional impacts of land use changes. Agricultural yields are also affected by these impacts, increasing the demand for more land for production, leading to the positive feedback [19]. As already demonstrated in Brazil, extensive land clearing for agriculture in the Cerrado and eastern Amazon alters regional weather, reducing maize yields by 6% to 8%, undermining the intended use of the cleared land [75].”

-P2, L29: clarify expansion: does this refer to an increase in crop area, or production, or demand?

**Response**: “Land use expansion for growing commodity crops...”

-P2, L48: does food just mean calories, or also micronutrients? (clarified on the next page)

**Response**: “Shifting diets towards those that contain lower quantities of livestock products could reduce the amount of land needed to produce food in terms of calories, macro- and micro-nutrients for human consumption.”

-P3, L65: need to support the statement that pasture has "marginal contribution to nutrient production".

**Response**: We clarified the sentence, added the following statement in the results section to show that most food nutrients are produced from crops and added the new supplementary file 5.

“Calcium and vitamin A for human consumption are mostly cattle based (up to 75% in certain years, as shown S1 Fig). Except for these nutrients, along with zinc and vitamin B12 (~30% to ~45% produced from pastures), more than 70% of the nutrients and calories are produced from food crops (S1 Fig).”

-P4, L134: R2 over different time periods seems like a weak basis for model selection.

**Response**: We used the adjusted R-squared to address the issue of overfitting when using R-squared with different number of variables. This was the case, in which we tested the period with better correlation in order to minimize the effect of abrupt changes in the trends over time.

-P7, L228: it’s problematic to say that crop calories increased threefold, animal calories increased fourfold, but this had minor effects on food availability. It may have had a minor effect on calories consumed domestically (which is largely a function of domestic population), but that is not the same as food availability.

**Response**: We do agree that the way we wrote led the authors to a misinterpretation of what we meant and made it clearer in the text now.

“Such an increase in crop production was mainly driven by export commodities with a less pronounced increase in food crops. The amount of food crop calories increased by 19%, from 1.40 x 10^14^ to 1.67 x 10^14^ kcal, while its proportion in total production declined from 32.8% to 13.0% (Fig 1).”

-P7, L241: the fact that Brazil exports 2/3 of the calories it produces makes it clear that domestic production capacity is not the constraining factor for domestic nutrient availability – as the authors note on P8, L280, and which should not be a surprise to most readers.

**Response**: This reinforces what is discussed later in the text that food insecurity is a matter of food access and that land use changes are not necessary to meet the nutritional requirements if food is properly distributed.

-P8, L269: the fact that there is no change in number of people fed (in terms of calories) reinforces the point that calorie consumption is closely correlated with population, and signals that consumers switch to higher value foods (in terms of price and nutrients) as their incomes rise.

**Response**: What is shown in this argument is that food production increases as population also increases and not that people are switching to more caloric diets. We clarified in the text:

“This provides evidence that the agricultural sector as a whole is shifting to market-oriented commodity production while the food calorie increases at the same rate of the population.”

-P8, L280: “food production was not the major issue for food security in Brazil”, neither in the future… At L296 the authors correctly note that food security in Brazil is a matter of access, stability and utilization – which, it should be noted, are not addressed in this paper.

**Response**: We strictly focused on the production side of food security, as food availability is the first constraint for food security. We clarified our focus with a revised title and in the introduction section:

“Focusing more specifically on the production side of food security (also referred to as food availability in food security literature), we combined supply and demand for calories, macro- and micro-nutrients based on three decades of data on agricultural production and trade, land use and nutritional requirements of the population.”

-P8, L301: are projected needs based only on changes in population, i.e. no change in income, prices, preferences, productivity, etc? If these important factors are left out, this needs to be noted explicitly, with a discussion of what difference that makes to the results.

**Response**: Addressed in the methods and in the discussion sections, respectively:

“Caloric and nutrient demand were calculated on the basis of the population projections and did not consider changes in income or individual preferences.”

“Moreover, rapid increase in food prices without increase in income or purchasing power is left out due to the limited scope of the analysis. However, our results indicate that proper food policies to buffer these side effects on food security are essential, once, as noted above, food production should not be a constraining factor for achieving food security in Brazil.”

-P9, L321: was maintaining trade mentioned earlier as a specific goal/constraint? Also note “feed its population” here seems to mean (just) providing sufficient calories and micronutrients (acknowledged on P11, L424).

**Response**: International trade was not the focus of the work, so we meant that even using 10% less land, enough food nutrients were produced even excluding the exported nutrients.

“In the past, Brazil could have used 10% less land to produce enough calories and nutrients for domestic supply (BAU). If food was properly distributed, even using less land for food production, would not compromise Brazilian agricultural exports.”

-P11, L387: diets shifting towards more meat: need to consider why (e.g. incomes and preferences), and what that means for measures to shift diets towards less meat.
**Response**: Acknowledged in the Discussion:

“The analysis of [63] reveals that income plays an important role in this pattern, not because of individual preferences to consume more meat, but due to the affordability of animal sources of protein. Strengthening food security policies that ensure the access to more diversified and fresh food could play an important role in shifting diets towards less meat consumption.”

-P11, L399: also reducing losses and waste.

**Response**: Addressed in the text.

-P11, L429: not clear.

**Response**: “Thus, considering the balance between production and demand of vegetables, cereals, meat and other food types could provide another approach of specific demand of land for each type of food product. For example, [68] considered the production and demand of vegetables at the municipality level in Brazil, providing information where vegetable sources are insufficient to meet regional needs.”

-P12, L454: demand not the same as needs (also food security in L455).

**Response**: “We highlight that Brazil holds a unique condition for delivering food security, (addressed here strictly in terms of nutrient availability), while limiting its land use expansion and promoting sustainable intensification of food production.”

*Reviewer #2*

This paper analyses agricultural land-use in Brazil by mapping land use for agricultural exports, comparing food production to nutritional requirements, and calculating scenarios for hypothetical diet changes. The paper is separable into two parts: the mapping of land-use and a comparison of agricultural land use with nutritional requirements. Both parts of the paper are based on appropriate data and provide the basis for a relevant and scientifically sound analysis. However, both parts would substantially profit from further refinements as described below. For the mapping exercise the current methodology is appropriate and does not require changes. The comparison of land and nutrient requirements needs to address methodological concerns, especially regarding the substitution between different agricultural commodities. As the interpretation of the two parts is not dependent on each other, I suggest that the authors split the work into two separate articles and resubmit the new papers. This would make better use of the large amount of work that appears to have gone into the paper and the scientific and policy potential visible in it.

In the first part, the authors use a rich dataset of municipality level agricultural production information and combine this with satellite data to produce a detailed map of land use for the production of different agricultural commodities. This detailed data is used to create a map of exported calorie shares, showing where most land is used for exports. This part of the paper makes good use of the detailed data. The authors do not analyze the map further and do not compare it with other geographical distributions. It would be very interesting to a) compare this map with maps of biodiversity or other measures showing the potential damage of land use changes and b) compare it to other relevant indicators like the economic value of the products. Comparing between these different layers of the map would help to show the trade-offs that certain agricultural policies require. This would give the work of the authors a much higher policy relevance.

The second part of the paper does not use the geographical details of the data but uses national aggregates of nutrient production and agricultural land use. The authors compare food production with the nutrient requirements of the population and apply different scenarios to show how much of the area would be required to meet the requirements. The authors differentiate between calorie requirements and a wider set of macro and micronutrient requirements. Using demographic projections, the authors show historic and future land requirements under the different scenarios. The topic and general approach of this part is interesting but it requires some alterations to the approach to provide practical relevance. My main concern is that the authors assume no change in the land use and the crops grown when shifting to a different diet. This would mean that consumers substitute meat consumption with the direct consumption of the respective feeds. Beyond being a very unlikely scenario, this has strong implications for the results of the study. E.g. the current paper concludes that the production system would not provide sufficient vitamin A under a vegan diet. A substitution of livestock products with fruits and vegetables instead of feed crops would most likely address this. Further, the authors leave the topic of agricultural exports unaffected. This is a reasonable simplification but it would be good to clarify this a bit more. Additionally, the methodology used to set nutrient requirements could be strengthened. The authors should shortly explain why they use the recommended dietary intake as reference in their calculation of micronutrient requirements while population level analyses of nutrient adequacy usually rely on estimated average requirements. Lastly, the requirements for all nutrients but calories are calculated from the demographic structure. The authors could do the same for calories, thereby harmonizing the approach.

**Response**: We are grateful for the valuable considerations to enhance the work we have done.

After a careful evaluation, we decided to remove the spatially explicit analysis from our results. We understand the suggestions of the reviewer and agree that we could make a better discussion of the spatial patterns of food and agricultural commodity production in Brazil. As this is not the main scope of the present manuscript, we made the following changes:

-Made clear in the methods that MapBiomas was used to retrieve yearly pastureland area and removed the subsection “*Mapping food and exports*”, which was strictly related to the maps:

“Additionally, we obtained crop and pastureland areas for every year from 1988 to 2017 from MapBiomas’s land use and land cover maps (collection 3.0). MapBiomas is a partnership of private companies, universities and NGOs that use all available series of remotely sensed images from Landsat satellites to produce land use and land cover maps for the Brazilian territory, in the period of 1985 to 2017, with 30 metres of spatial resolution [39]. Based on the municipal data from PAM, the class “Crop or Pasture”, an error class in MapBiomas that cannot be distinguished due to spectral confusion, was allocated to “Pasturelands” or “Croplands” classes, adjusting the cropland maps to the official Brazilian cropland area. This allowed us a yearly estimate of the pasture area at the national level.”

-Removed the maps (Figure 2) and the following paragraph:

“Currently, most of the crop calories that are produced in the north-east and in the extreme south regions of the country are produced as food (Fig 2), while those produced in the centre-south are exported. A similar pattern, with less pronounced division, was observed for 2003, when Brazil consolidated as a net exporter of crops [48]. In the beginning of the study series, 1988, a very different distribution was found, when higher proportions of national crop production were used for food (Fig 2).”

After these changes, we considered the commentaries about the argued “second part”. We highlighted in the Introduction that land use expansion for food production is often argued as necessary in policies to weaken environmental protection in Brazil. The basis of our work is to investigate whether the current land use could provide sufficient nutrients for the Brazilian population. This is the main reason to assume no change in the land use.

“the current paper concludes that the production system would not provide sufficient vitamin A under a vegan diet. A substitution of livestock products with fruits and vegetables instead of feed crops would most likely address this.”

We conclude that the current production system would fail in provide sufficient vitamin A under a vegan diet. This is why we recognize that “A full assessment of food and nutrition security will require consideration of such aspects”, which is at the scope of our future investigations.

Regarding exports, we left unchanged because our focus is on the domestic food production. Our results show that even without changes in the exports, less land could be used to achieve the nutritional demands of the population.

Finally, we added the following sentences in the methods to clarify the confusion about RDA and caloric calculations:

“According to the United States Institute of Medicine, there is no RDA for energy because energy intakes above the Estimated Energy Requirement would be expected to result in weight gain [42]. Thus, the proportion of the population potentially fed with national food production was estimated on the basis of 2,450 kcal/person/day diets (894,250 kcal/yr), which is the maximum value of Average Dietary Energy Requirements (ADER) recommended by FAO for Brazil.”

[…]

“Following the methods from [50], whenever available, we use the Recommended Dietary Allowance (RDA) which represents the average intake that would meet the needs of 97.5% of healthy individuals in a group. If an RDA was not available for certain nutrients, we used Adequate Intake (AI), which is the level of intake assumed to be adequate for healthy individuals. The daily requirements for each nutrient in each year, weighted for the Brazilian population, is found in the S2 Table. This way, we had both nutrients and calories averaged for the structure of the Brazilian population every year in the period of analysis. Caloric and nutrient demand were calculated on the basis of the population projections and did not consider changes in income or individual preferences.”

Minor comments:

1) The authors should provide supporting arguments for the assumption that all cattle is grazing (line 121) or adapt the calculation. Especially for dairy production, single reliance on grazing appears questionable.

**Response**: We apologize and recognize that this sentence leads to a misinterpretation of our assumptions. We did not mean that dairy production relies only on grazing, as clarified in the line 176-177 of the first version of the submitted manuscript. We meant that we did not account for dairy under confinement systems, that corresponds to only 2.4% of the production in Brazil. Thanks for the advice.

“We assumed that both beef and milk cattle were produced in grazing and not confinement systems, which accounts for only a small fraction (2.4%) of cattle production in Brazil [77].”

2) The authors could consider to introduce the quoted inequality in the food distribution into their calculation of required nutrients. This would mean to calculate requirements under the assumption that the current distribution remains unchanged. This suggestion is optional and authors might choose the current simplification.

**Response**: We made a brief statement in the Discussion to address the important issue of inequality:

“The causes of food insecurity in Brazil are likely to be rooted in poverty and in the inefficient distribution and retailing of food, that constrains stable access to healthy food. Between 2013 and 2018, mild food insecurity increased by 71.5%, while severe, which characterizes hunger, increased by 48.8%, totalling around 10.3 million people in food insecurity in Brazil [78]. Even while exporting more agricultural commodities and producing enough food, Brazil is in danger of again being reinstated on the world hunger map.”

3) Formula (1) contains XY as index but the explanation does not explain what these are. Also, the term fcrop is described to be “the map of a certain crop area”, which is unclear. I assume that this might refer to the state of the specific grid cell. I suggest to fully review the formula and its description.

**Response**: Excluded from the manuscript as part of the spatially explicit analysis.

4) Figure 1 and/or its description are unclear. E.g. It is unclear if the dotted and dashed lines are part of the solid lines and why animal and crop calories proportions in some years sum to more than 100% of all produced calories and in other years to less.

**Response**: Clarified in the description.

“**Caloric production in Brazil from 1988 to 2017**. Animal (red) and crop (green) calories sum 100% each, disaggregated into calories for export (dashed lines), domestic food consumption (solid lines) and animal feed (dotted lines).”

5) Replace the term “food system” with “domestic demand” or a similar turn to avoid confusion with the food systems literature (e.g. line 244).

**Response**: Addressed in the text.

6) The description of figure 2 refers to the graphs in the wrong direction.

**Response**: Excluded from the manuscript as part of the spatially explicit analysis.

7) Line 296f. refers to food stability. It is unclear what this term means.

**Response**: Stability is how food is produced, accessed and used in time. We found that the word is redundant in the sentence and removed it.

8) Line 306 appears to use “with” instead of “without”.

**Response**: Addressed in the text.

9) In line 403ff. it is unclear what kind of estimates this refers to and why no clear number is given (“circa”).

**Response**: Clarified in the text.

“based on estimates of circa the year 2000 (1997-2003).”

10) Lines 415 ff. refers to income deciles representing “less than 1% of the population” while a decile per definition is 10% percent of the population.

**Response**: This refers to income deciles instead of population deciles. In this case, 1% of the population account for the 10% higher income.

11) I suggest to carefully review the writing and style: At a few points punctuation (e.g. in the abstract), letters, or words (e.g. line 3) are missing or too much (e.g. line 205, line 273, caption of figure 3, line 312). In line 47 the term ”on the other hand” is used but the sentences do not describe contradictory arguments. The paragraph from line 395 repeats the term required five times, where it would read better with the usage of other terms. The sentence in lines 431ff. is incomplete.

**Response**: Thanks for the advice. We have corrected typos and checked for minor spelling errors.

12) Line 65 describes the nutrient supply from beef as marginal. Based on the large quantity of specific nutrients (e.g. vitamin B 12) produced for export and domestic use (as visible in the authors’ own calculation) this seems not to be fitting. It would be better to make an efficiency argument regarding the nutrients produced per hectare. I further recommend to split the sentence.

**Response**: We clarified the sentence, added the following statement in the results section to show that most food nutrients are produced from crops and added the new supplementary file 5.

“Calcium and vitamin A for human consumption are mostly produced in pastures (up to 75% in certain years, as shown S1 Fig). Except for these nutrients, along with zinc and vitamin B12 (~30% to ~45% produced from pastures), more than 70% of the nutrients and calories are produced from food crops (S1 Fig).”

13) The link on reference 42 does not work.

**Response**: The following link is corrected:

https://www.nal.usda.gov/sites/default/files/fnic_uploads/Framework_DRI_Development.pdf

14) Table S1 contains a group of crops, where it is unclear why these are selected. To make the table more useful it would be good if the authors could tabulate the numeric values, e.g. for each of the three mapped periods per crop or crop group.

**Response**: Clarified why only a few crops are listed:

“Crops omitted from this table were allocated exactly as reported in the FBS for each specific crop.”

15) In table S2 some units are wrong (e.g. gram of vitamin C per day).

**Response**: Thanks for the advice. We checked all the units and corrected this wrong one.

16) In table S4 it would be interesting to know if aquaculture is excluded. Also, the description refers to agricultural expansion while the paper otherwise assumes that the land usage for each crop remains stable for this form of analysis.

**Response**: The major land use in Brazil is agriculture, especially for livestock. According to the Agricultural Census of 2017 (Table 6778), only 0.37% of the farms had any form of aquaculture and this is why we did not include it in our analysis.

17) The uploaded data contains the nutrients per municipality but not the information required to reproduced the map for exports or the land usage per grid cell. This would not allow to reproduce the results.

**Response**: Excluded from the manuscript as part of the spatially explicit analysis.

*Reviewer #3*

The objective of the reviewed article is ostensibly to understand the capacity of the Brazilian agriculture system to provide for food security at the country/national level. The basic question, it seems, is to understand whether or not there is sufficient capacity in the Brazilian agriculture system to feed the population now and well into the future. The premise is reasonable and there are findings presented in the paper that could support decision making related to food system policy.

While there are some novel elements to the paper, there are a number of issues - some significant, some modest - that need to be addressed before the paper is ready to be published.

The first issue of concern is simply the core thesis of the paper. Is it that Brazil has sufficient agricultural capacity to provide for food security, or lower levels of meat consumption are the required in order for Brazil to achieve food security? I ask this as the the former seems to get obfuscated by the latter in several places in the paper. Likewise, related to the same, there have been a surge of recent papers and reports (the EAT Lancet Report, for example) that have unpacked similar issues at the global scale. There is a lot of potential context around the relationship between food security and planetary boundaries that could be considered in relation to the present effort, but much of this literature is omitted from the current manuscript. Another area of concern is the authors are emphasizing food security in general terms but really only address the issue of food availability (at least in relation to the problem statement - other issues come out in the discussion, but this again leaves me wondering about the key messages and question of this work).

A second area for consideration is in relation to the relatively complex dynamics that happen with regard to prices, land use, trade, etc., as the structure of agriculture production shifts. The authors make a number of simplifying assumptions (many of them reasonable, I might add), but largely treat the question of agriculture production as an economic isolated construct. From a food system perspective, it is critical to consider the different trade-offs that may come with different policy decisions (e.g., perhaps policy to reduce meat consumption). Aspects of this are loosely acknowledged in the MS, but only generally (e.g., lowering the Brazilian food footprint while maintaining trade in animal products); that said, these issues are highly complex and merit additional consideration given the way in which the argumentation is made in the paper.

The issues mentioned above also have significant implications for the future scenarios. There are several scenarios discussed relating to changing diets (specifically towards lower meat consumption) and land use. As mentioned above with regard to the central thesis, the precise justification of the scenarios does not come across as totally clear. Is this an arbitrary choice, is this related to a policy objectives, is this consistent with the recent planetary boundaries work, etc?

While the inputs are clear and certain key aspects of the methods are clear (e.g., the specific periods associated with the projections), the methods as a whole are unclear in terms of the whole and how each step/part links to the central thesis. One relatively clear example (at least for this reviewer) is the use of the MapBiomass data. There seems to be a fairly significant disconnect between the highly detailed MapBiomass analysis and the later projections forward in time. Again, this might connect back to the needed to more crisply define the objective and specific research question associated with the manuscript.

There are some interesting results and the authors should consider how they help focus the central premise of the paper. For example, they talk about national caloric production and then growth of population. Would this be better expressed in per capita terms given the emphasis on food security?

Finally, I am not sure if the confidence analysis adds much support. Part of this stems from the lack of clarity on certain aspects of the analysis. Given the extrapolative nature of the study, it seems there should either be systematic characterization of each of the analytic steps for verification and validation and/or some sort of sensitivity analysis associated with the overall model results.

While challenging, none of the above are insurmountable, and I would therefore recommend major revisions and a substantial clarification on both the thesis and its basis before the paper is ready for publication.

**Response**: We thank the reviewer for the contribution to improving the manuscript. In general, the major comments were addressed in the responses to the other reviewers. Some specific topics related to the comments are the following:

- We improved the introduction to show that our aim is to explore if the current land use, along with different dietary patterns, would be enough or more land is needed to produce more food. The need for agricultural expansion is often claimed by policymakers in Brazil and our results point in the opposite direction. Neither dietary nor land use changes are needed to supply the food nutrients for the population. In addition, dietary changes could save a considerable amount of land, also pointing to the fact that no agricultural land use change is needed in Brazil. We recognize that diets should contain large amounts of fresh and diversified food, but this is not at the scope of this analysis.

At this point, the highlighted parts were added to the main text:

“Despite law enforcement to reduce deforestation in Brazil from 2004 to 2012, significant changes in the environmental legislation were made under pressure from the agribusiness sector [30]. At the same time, the government reduced its public budget for environmental actions and benefited agribusiness expansion with financial support. This resulted in increasing deforestation trends since 2012, reaching in 2020 its highest peak since 2008 [27–29].

Political efforts to reduce environmental protection in Brazil were often based on the argument that more land is needed to produce food to the Brazilian population [30-32]. This argument was central in the debate that changed the environmental legislation in 2012 and is still argued in some proposals, both from the national congress and government, for promoting the agricultural expansion [30]. However, the proportion of land needed in the territory for meeting the nutritional food demand of the Brazilian population, currently around 210 million people, is unknown. [31–33] argue that increasing the productivity of pasturelands could negate the need for any further land to be deforested land for food production. Additionally, [34–36], point to the importance of considering that more food can be delivered on the same amount of land if dietary preferences are changed. Enriching diets with plant-based foods and with fewer animal sources is argued to be beneficial for both human health and for the ecosystems [76], reducing the environmental pressures of food production on the environment. Shifting diets towards those which contain lower quantities of livestock products could reduce the amount of land needed to produce food, in terms of calories, macro- and micro-nutrients for human consumption. Taken together, increasing yields through sustainable intensification and changing diets could make a significant contribution to deliver food security while reducing the pressure on the environment due to land use expansion.”

- We focused on the land use as deforestation for agricultural expansion is currently one of the key environmental issues in Brazil. A full assessment of trade-offs also requires broader evaluation of ecosystem services which is not our main objective here.

“Given the importance of Brazil for the provision of key global and regional ecosystem services, the trade-offs between agricultural production and environmental impacts should be further investigated, specially in the scope of the planetary boundaries discussion.”

- Food production is a central political argument for land cover changes in Brazil. This is why we assumed no land expansion.

“We used 2017 food production as the baseline for the future projections, assuming that any additional land is used after this year. This simple assumption is adopted to test the claim that more land is needed to feed the Brazilian population.”

- Regarding MapBiomas, we followed the suggestion of other reviewer and excluded the spatial explicit results from the current manuscript. We did maintain the pastureland areas calculation from MapBiomas, as explained in the Methods.

“Additionally, we obtained crop and pastureland areas for every year from 1988 to 2017 from MapBiomas’s land use and land cover maps (collection 3.0). MapBiomas is a partnership of private companies, universities and NGOs that use all available series of remotely sensed images from Landsat satellites to produce land use and land cover maps for the Brazilian territory, in the period of 1985 to 2017, with 30 metres of spatial resolution [39]. Based on the municipal data from PAM, the class “Crop or Pasture”, an error class in MapBiomas that cannot be distinguished due to spectral confusion, was allocated to “Pasturelands” or “Croplands” classes, adjusting the cropland maps to the official Brazilian cropland area. This allowed us a yearly estimate of the pasture area at the national level.”

- We use many sources of data to calculate how much nutrients the population in a given year requires and how much is produced. We presented the results in percent of the population that could be fed with the domestic production. This is found in the literature as people fed and/or nourished per hectare, as explained in the Methods. In other words, it means per capita production per hectare.

There are also a number of minor errors and style issues in the document and the manuscript would benefit from careful proof reading in advance of resubmission. For example, please see:

Line 61 - Our study provideS...
Line 153 - nutrinional - should be nutritional

**Response**: Thanks for the advice. We have corrected typos and checked for minor spelling errors.
