# Supplementary material for: Is domestic agricultural production sufficient to meet national food nutrient needs in Brazil?
Source: PLoS One. 2021 May 20;16(5):e0251778. doi: 10.1371/journal.pone.0251778 (PMC8136643; doi:10.1371/journal.pone.0251778)
Supplement: Attachment — Submitted filename: Response_to_Reviewers.doc [file pone.0251778.s007.doc]

**Response to Reviewers**

-We are grateful for the previous and current advices made by the reviewers, as well as the kind consideration of the editor. They are essential to strengthen the paper and to make our results and its implications accessible to a wider audience. We have carefully addressed all the issues raised by the two anonymous reviewers, as follows. Finally, we have gone through a careful spell check by native English speakers.

*Reviewer #1*: The paper is much improved with greater focus on the core question of domestic production and nutrient needs. A bit more clarification would help sharpen its main finding. Specifically, a key motivation for the paper is to counter the argument that environmental protection measures should be reduced to increase food production for the Brazilian population (line 87). The paper succeeds in doing this. But the paper then needs to note more clearly that Brazil’s importance as an agricultural exporter (lines 59, 258) suggests that export earnings are perhaps a more critical factor behind the argument for reduced environmental protection. This raises further questions about how such earnings are distributed. Line 442 notes that such earnings can improve food security for some people, but line 444 indicates these benefits may be offset by unspecified adverse social impacts, possibly including increased inequality (line 440). It would help strengthen the paper’s conclusions if the authors would come back more explicitly to what seems to be the primary argument for expanded agricultural production – namely continued growth in export earnings.

*-Response*: We thank the reviewer for the encouraging commentaries and for the further suggestions. We do agree with the point raised here and recognize that it is worth mentioning it, as we did. In order to avoid a longer, we added in the paragraph that refers to the impacts of the exportation of agricultural commodities: “This reflects that most of the Brazilian agricultural exports in volume are raw products with low added value, like coffee and soybeans. These products account for nearly a fifth of the country’s GDP, with increasing trends. Therefore, the continued growth in export earnings seems to be the primary reason for expanded agricultural production rather than improving the people’s food security.[...]”

*Reviewer #2:* The authors addressed the major shortcomings and suggestions previously made. The article provides an interesting case against arguments that excuse land transformation in Brazil with references to domestic food security. With the changes to the manuscript this argument becomes more clear and it will appeal to a wider audience. I appreciate the authors’ answers to the previous comments, which addressed major concerns and clarified the limitations of the analysis. Only minor issues remain to be addressed in the article.

Minor comments: There are several language errors and a number of sentences are very long or wordy. I marked a number of errors and made specific suggestions below. However, these are not necessarily complete. The article could profit from additional language editing.

With regards to the terminology, I suggest being careful with the usage of the term “food demand”. A specific point is made in the comment below.

There are errors in the numbering of the citations. They are not numbered in sequence of their first citation.

Below, please find the detailed comments below:

Abstract: The authors could consider deleting the following two sentences as they are repetitive with the well explained results in the two sentences before: “We conclude that the Brazilian agriculture could deliver enough food for meet Brazilians’ nutritional needs without any additional land or changes in current diet. Nevertheless, our results show that shifting to diets with lower levels of meat consumption would substantially reduce land demand for feeding the population.” A shortened version referring back to the second sentence could be: “We conclude that no land expansion is required to produce sufficient nutrients for the Brazilian population. Food production is compatible with environmental conservation […]”

(line 42 with track changes) “Reducing the impacts of the agriculture on the environment [..]” or “We conclude that the Brazilian agriculture could deliver enough food for meet Brazilians’ nutritional needs […]” (line 57f.).

- We removed the sentences and rewrote the idea as follows: “We conclude that Brazilian agriculture could deliver enough food to meet Brazilians’ nutritional needs without further land expansion. Food production is compatible with environmental conservation in Brazil, especially if meat consumption is reduced.”

Line 42: Should be “Reducing the impact of agriculture on the environment [..]”

-Addressed in the text.

Lines 47-54: I suggest to separate the sentence and put the statement on GHG emissions in a second sentence.

-Addressed as follows: “Expansion and intensification of agriculture since the 1960s was successful in augmenting food production, but it also caused numerous adverse environmental impacts [3]. Important impacts from agriculture, though not limited to these, are habitat loss and fragmentation, with almost 40% of the ice-free land surface used for agriculture [4], growth in nitrogen fertilizer and water use of 800% and 100%, respectively [5] and emission of 23% of the total anthropogenic Greenhouse Gases (GHG). Especially, GHG emissions contribute to the current increase in global average air temperature”.

Line 57f.: “[…] enough food to meet […]”

-Addressed in the text.

Line 59: “70% of global trade”, without “the”.

-Addressed in the text.

Line 60: For readability, I suggest: “[…]global land cropped for the international trade in agricultural commodities. This makes it the third largest exporter in the world, after the European Union and the United States and highlights its role in the global food system.”

-Addressed in the text.

Line 63: Split into two sentences: “biodiversity. It has the largest tropical forest area and carbon stock in the world, thereby contributing to […].”

-Addressed in the text.

Line 70, 74: The numbering of the citations 74 and 75 is wrong. (A few lines later the same problem repeats with number 30).

- These references were inserted after the first revision and, therefore, the numbering is not continuous along the text.

Line 83-84: The sentence is unclear. Maybe: “Despite the introduction of legislation to reduce deforestation in Brazil […]”

- We think it is important to clarify that not only legislation, but government actions were important for reducing deforestation rates. Thus, we adapted the suggestion to: “Despite the introduction of legislation and governmental actions to reduce deforestation [...]”

Line 84-86: I suggest to replace “benefited” with “encouraged”.

-Addressed in the text.

Line 107: For terminological reasons, I suggest to avoid the word “demand”. Demand is defined by the purchasing power and preferences of consumers, while the article refers to the required nutrients. I suggest to refer to “dietary requirements” or a similar term that specifies that the discussion centers around physiological required quantities. (Also the Article of Depenbusch and Klasen (2019, https://doi.org/10.1371/journal.pone.0223188) has a short section discussing this difference.)

-Addressed in the whole text and referenced the suggested paper.

Line 120: “food and non-food uses”, using “and” instead of “or”.

-Addressed in the text.

Line 204: The sentence appears to have a mistake and it becomes very long. I think it should be “[…] Potassium), for which we had food composition data. This allows us to access […]”

-Addressed in the text.

Line 255f.: Please rephrase the following sentence to make it clearer: “Domestic food calories from animal sources is always above 75% of the total animal calories produced in the period.”

- We rephrased the sentence: “On the animal production side, more than 75% of the calories are for food, in the entire time series.”

Line 257: The term “all of each from dairy” is unclear. Do you mean that all animal calories used for feeds are from dairy?

- Yes. We made it clearer: “less than 5% of animal calories, which are derived from dairy products.”

Line 261-263: I would suggest to split the sentence to make it easier to read: “In 1988, calorie exportation (both plant and animal) was equivalent to 27% of all food calories. Since 2002 food calorie exports surpass domestic consumption.”

-Addressed in the text.

Lines 265-267: Looking at the graph, it seem that crop calories do not sum up to 100%. Could this be because losses and other usages are not plotted? If so, I recommend to just clarify this in the caption.

- Thanks for the advice. “Animal (red) and crop (green) calories sum 100% each, disaggregated into calories for export (dashed lines), domestic food consumption (solid lines) and animal feed (dotted lines). Wastes and other uses are not shown.”

Line 286ff.: Suggestion for better readability: “However, as only a fraction of these calories are available as food, it suffices for less than one person per ha. Considering the production of plant and animal food products in the time span between […], on average 0.96 (±0.06) persons could be fed per ha and year.”

-Addressed in the text.

Line 292: Spelling should probably be “[…] at the same rate as the population”. It is interesting to see that international trade leaves just a bit less calories in the country compared to what is required. This could be misinterpreted as a situation where enough is provided for every person. As you rightly argue, due to unequal access to food this is not the case. It might be worthwhile to quote the 2020 SOFI of FAO. In Table A1.1 it specifies that about 1/5 of the population suffers from moderate or severe food insecurity.

- We corrected the spelling. To make the readers aware of the inequality and to avoid discussions in this section (as suggested below by the reviewer regarding food fortification), we added the following sentence in red: “This provides evidence that the agricultural sector as a whole is shifting to market-oriented commodity production, while the food calorie increased at the same rate as the population. As discussed below, however, increasing food production does not reflect an even distribution of food for every person.” Also, we noted in the discussion that according to FAO, 1/5 of the population suffer from moderate or severe food insecurity: “This is illustrated by the fact that between 2014-16 and 2017-19, the number of moderately or severely food-insecure people in Brazil increased by 15%, reaching a fifth of the population [78].”

Line 294ff.: This paragraph sums-up the provision of micro and macro nutrients but after this the new chapter starts and gets back to the historical micronutrient consumptions. I suggest to consider copying lines 301 to 316 before this paragraph. Then the following chapter focuses purely on the scenarios and not on historic values. This might also make it easier to distinguish between the produced and the domestically available micro nutrients. Currently the lines 301 ff. are a bit confusing on that point, talking about Vitamin A as the most restricting nutrient, while it still suffices for a larger population share than that quoted in lines 294ff..

- We have merged the sections “Macro- and micro-nutrient production” and “Diet scenarios” and divided the paragraph to address this suggestion. We believe that this new format improves the clarity of this part. Thanks for the advice.

Line 299: Delete empty line.

-Addressed in the text.

Line 311ff: I suggest to rephrase the sentence “In turn, since 2009 the produced vitamin A was sufficient for more people than the produced calories.”

-Addressed in the text.

Line 314: I suggest to avoid the term “demand”, maybe use “dietary requirements” here.

-Addressed in the text.

Line 335: Typo (“de”)

-Corrected

Line 338: “by comparing the curves”, without the additional “the”.

-Corrected

Line 341: I think “huge difference it has with a no-beef diet” should be replaced with “huge difference to a no-beef diet”.

avoid the term “demand”, maybe use “dietary requirements” here.

-Addressed in the text.

Line 350f.: The sentence in parentheses has twice the word “but”.

-Corrected

Line 357f.: This seems to contradict the provision of 99% of adequacy quoted in line 294ff. for Vitamin A. Please clarify this.

- In line 294, we referred to 0.99 people nourished per hectare (i.e. 99 people from 100 ha). We clarified this by changing pp/ha by caput/ha.

Line 364f.: I suggest to just quote the inadequacy of Vitamin A and B12 here and discuss fortification as one option in the discussion.

- We removed the sentence “ which would require fortification and supplementation of these nutrients for an adequate vegan diet” and added in the discussion: In the BAU scenario, 220 Mha are required to produce food required by 2060, while completely shifting the diets towards a vegan diet would require only 14.5 Mha, despite limitations in vitamins A and B12 availability. Here, food fortification would play an important role for meeting the nutritional requirements of the population. Also, enhancing crop production, closing yield gaps, reducing losses and waste are strategies to deliver higher yields and increase food availability using less land [35, 65]. The redesign of the agricultural systems is critical to limit land requirements and potential expansion over natural ecosystems, regardless of dietary change.

Line 394: I suggest to shorten the sentence to improve readability “[…] by [31], who used the same sources.”

-Addressed in the text.

Line 397: Delete empty line.

-Addressed in the text.

Line 402-404: Kindly check the definition of food security in the quoted source. The FAO definition, based on the Food Insecurity Experience Scale (see page 193f. in the 2020 SOFI). By that definition, the association with “hunger” would be less clear and I would suggest to drop the reference to it in explaining food insecurity.

- We quoted the national survey that is reported to FAO, where hunger is also considered. To avoid misinterpretation, we changed the citation to FAO’s SOFI as follows: “This is illustrated by the fact that between 2014-16 and 2017-19, the number of moderately or severely food-insecure people in Brazil increased by 15%, reaching a fifth of the population [78].”

Line 406: I think it is more appropriate to say that the consumption of highly processed foods is a major determinant. You could for example rephrase: “As we focus on the production of food crops, we ignore later stages of the supply chain and the food environment, which have an important effect on the way food is consumed (e.g. through the consumption of highly processed foods).”

-Addressed in the text.

Line 408: The word “about” can be dropped.

-Addressed in the text.

Line 409: “natural resource preservation” without “s”

-Corrected

Line 418: “[…] protecting the country’s […]”

-Corrected

Line 418-419: The wording seems wrong “This is a complex issue that can be approached in multiple ways.”

-Addressed in the text.

Line 420: Wording seems wrong: “[…] can have a sizable impact on land demand for pastures, […]”

-Corrected

Line 421: The wording seems off and a bit long. I suggest “On the production side, pasture productivity enhancement and optimised cattle supporting rates [31, 32] can reduce the demand for pastureland.” This more careful wording would consider that productivity enhancing steps alone cannot ensure a decline in land transformation. The data of the authors shows this very nicely.

- Thanks for the advice. We agree and addressed this comment in the text.

Line 431: Please clarify if this is just about international trade in agricultural commodities.

- Clarified: “Regarding export of agricultural commodities, Brazilian international trade has higher shares of calories (9%–10% of global trade flows) than of monetary value (4%–5%) [10].”

Line 432-433: I would rather say that it “reflects” the high share of raw products, as there is no strong reason why the ratio between calories and monetary value should be strongly correlated.

- Thanks for the advice. We addressed in the text.

Line 448: I think it should be “are” not “and”.

-Corrected

Line 450ff.: Please rephrase the sentence and split it into two. It is hard to read and has some errors at the moment.

- We rewrote as follows: “Here, food fortification would play an important role for meeting the nutritional requirements of the population. Also, enhancing crop production, closing yield gaps, reducing losses and waste are strategies to deliver higher yields and increase food availability using less land [35, 65]. The redesign of the agricultural systems is critical to limit land requirements and potential expansion over natural ecosystems, regardless of dietary change.”

Line 456: Before you use “p.p.” to say per person, here it is “caput”. (the same in lines 469ff.)

- We now refer only to “caput”.

I suggest to shorten the paragraph to one or two sentences, just stating that the differences are due to the exclusion of pasturelands and the inclusion of exported calories in source [36].

- We addressed as follows: “To compare with [36], we found quite similar values for the year 2000 (12.2 and 3.3 caput/ha, respectively). The differences in our estimates are due to the exclusion of pasturelands and the inclusion of exports in their analysis.”

Line 457: “[…] based on estimates over the period 1997-2003.” Please clarify (e.g. in the previous sentence) why you refer to the year 2000.

- This corresponds to the data availability for the study of [36], as stated in the text: “The estimates of [36] are based on data of circa the year 2000 (1997-2003).”

Line 490: The word “they” is missing in “[…]although they have[…]”.

-Corrected

Line 500-504: The connection to incomes has been discussed before, so I suggest not to discuss it here again as shortcoming.

- Thanks for the suggestion.

Line 507f.: “Finally, we did not consider the role of food imports one land demand and our diet scenarios.”

-Addressed in the text.

Line 514ff: I would suggest “We provide evidence that the food and nutritional needs of the Brazilian population do not necessitate an expansion of agriculture land beyond its current range.”

-Addressed in the text.

Line 520ff.: I suggest to delete the term “Additionally, public awareness for” to be less wordy.

-Addressed in the text.

Line 522: The sentence got an error. I suggest “This would allow to maintain key […]”

-Addressed in the text.

Lines 523-526: The sentence got a language error. Also soil conservation (though connected) has not been discussed before. Looking into sustainable agricultural practices opens a wide new area of possible policies and interventions. I suggest to either point towards them more generally or dropping the reference here.

- We opted to drop the sentence to avoid further enlargement of the discussion section.

References: Please add DOIs, where possible. Please check that the sources are quoted according to the journal standards. In particular, please correct following sources and add missing information (e.g. publisher, place published for books): 7, 13, 46, 57, 67. I recommend to use a citation software, if not done before (e.g. Zotero).

- DOIs and links were added to the references. The mentioned references were corrected.

Line 738: I think the language is wrong. I suggest “Assumptions underlying the scenarios”

-Addressed in the text.

Table S2: The quantity for micro gram (μg with the Greek letter μ) has been replaced with ug. If the Greek letters do not work you could use mcg. Also please note for Vitamin A as unit RAE (retinol activity equivalents), behind the term for mcg, as is convention.

-Addressed in the table.

Table S3: I think the word “Any” should be replaced with the word “None” in this context.

-Addressed in the table.
